# The expression of miR-211-5p in atherosclerosis and its influence on diagnosis and prognosis

**DOI:** 10.1186/s12872-021-02187-z

**Published:** 2021-08-02

**Authors:** Yanxia Zhang, Huiyun Wang, Yu Xia

**Affiliations:** Department of Health Comprehensive Geriatrics, Yidu Central Hospital of Weifang, No. 4138, Linglongshan Road, Weifang, 262500 Shandong China

**Keywords:** Atherosclerosis, MiR-211-5p, Diagnosis, Prognosis

## Abstract

**Background:**

The purpose of this study was to evaluate the diagnostic and prognostic significance of miR-211-5p in atherosclerosis (AS) by detecting the expression level in serum of patients with AS.

**Methods:**

A total of 85 healthy controls and 90 asymptomatic AS patients participated in this study. The expression level of miR-211-5p in all subjects were measured by qRT-PCR. Spearman correlation coefficient was used to evaluate the correlation of miR-211-5p with CRP and CIMT. The ROC curve was established to assess the diagnostic value of miR-211-5p in AS. The Kaplan–Meier survival curve and multivariate COX regression analysis were used to evaluate the prognostic significance of miR-211-5p in AS.

**Results:**

The expression levels of miR-211-5p in AS patients were significantly lower than in healthy controls (*P* < 0.001), and miR-211-5p showed a significant negative correlation with CRP (r =  − 0.639, *P* < 0.001) and CIMT (r =  − 0.730, *P* < 0.001). The AUC of the ROC curve was 0.900, the specificity and the sensitivity were 84.7% and 78.9%, respectively, which indicating that miR-211-5p had diagnostic value for AS. Survival analysis showed that patients with low miR-211-5p expression were more likely to have cardiovascular end-point events (Log rank *P* = 0.013).

**Conclusion:**

Serum miR-211-5p could be used as a new biomarker for the diagnosis of AS, and the low expression of miR-211-5p is associated with the poor prognosis of AS.

## Introduction

Cardiovascular and cerebrovascular diseases, which are closely related to atherosclerosis (AS), are the main cause of death and disability worldwide. Since it is difficult to detect, the early diagnosis of AS is challenging. Therefore, early diagnosis of AS is a vital method to reduce disease damage [1]. Modern medicine believes that lipid metabolism disorder is the pathological basis of AS. The basic characteristics of AS is the accumulation of lipids and carbohydrates in the intima of arteries and the formation of thrombus, which leads to the proliferation and calcification of fibrous tissues, and ultimately to the thickening and hardening of arterial walls and narrowing of vascular lumen. Once the lesions has progressed to the point of blocking the arterial lumen, the tissues and organs supplied by the artery will become ischemic or even necrotic [2, 3]. Although the diagnosis and treatment of AS in human beings have made rapid development in the past few decades, the incidence of cardiovascular disease is still on the rise, and new methods or measures are urgently needed to overcome this disease. With the development of molecular pathophysiology and genetics, it has become possible to determine the occurrence and development of diseases by monitoring changes in genes [4, 5].

MicroRNAs (miRNAs) are a class of endogenous non-coding RNAs with regulatory functions found in eukaryotes, which regulate gene expression mainly by targeting mRNA for cleavage and translation inhibition [6]. miRNA regulates cell growth and tissue differentiation and is associated with life and disease development. In the cardiovascular system, miRNA controls the functions of various cells, such as cardiomyocytes, endothelial cells, smooth muscle cells and fibroblasts, providing new ideas for the study of cardiovascular diseases, such as myocardial infarction, arrhythmia and AS [7]. Studies have shown that miR-155 can regulate the expression of angiotensin II receptor, which is negatively correlated with miR-155 and positively correlated with blood pressure. In addition, the miR-155 is located on chromosome 21, and trisomy 21 syndrome is associated with decreased blood pressure [8]. Vascular calcification and senescence are common in patients with AS, and a study by Xu et al. showed that miR-211 in exosomes can inhibit calcification of the medium membrane of the vascular wall and senescence of vascular smooth muscle cells [9]. Another study confirmed that miR-211 expression was significantly down-regulated in serum of patients with calcified aortic valve disease compared with the control groups, suggesting that the disease may be associated with regulation imbalance of miR-211 [10]. Sara et al. reported that miR-211, miR-204 and other genes are involved in vascular smooth muscle cell calcification [11]. Zhang et al. reported that plasma miR-211 could be used AS a dynamic monitoring factor for the progression of diabetic AS [12]. Although the correlation between miR-211 and the occurrence and development of cardiovascular diseases has been confirmed in the literature, there are few systematic studies on the association between miR-211 and AS in relevant fields.

In this paper, the expression levels of serum miR-211-5p in all subjects, including 85 healthy controls and 90 asymptomatic AS patients, were examined to evaluate the diagnostic and prognostic value of miR-211-5p in AS.

## Materials and methods

### Study population and sample collection

The 90 asymptomatic AS patients and 85 healthy controls recruited in this study have excluded individuals with stroke, heart disease, angina pectoris, heart failure, hypertension, renal insufficiency, or other cardiovascular diseases. The carotid intima-media thickness (CIMT) of all subjects were detected by ultrasound, and individuals with CIMT ≥ 0.9 mm but < 1.2 mm were diagnosed as asymptomatic AS. In addition, physical examinations were performed on all subjects included in this study, their age, gender, height, weight, and other information were recorded, body mass index (BMI) was calculated. The blood of all subjects was collected, centrifuged, and stored at − 80 °C. This research protocol was approved by the clinical research ethics committee of Yidu Central Hospital of Weifang, and all methods were carried out in accordance with relevant guidelines and regulations. All participants have signed written informed consent.

### Quantitative real-time PCR analysis

TRIzol reagent (Invitrogen, USA) was added to the participant's serum to extract total RNA from the blood, and then the PrimeScript RT Reagent Kit (Takara, Tokyo, Japan) was added to reverse transcribed RNA into cDNA. Using cDNA as a template, qRT-PCR analysis was performed according to the instructions of the SYBR Premix Ex Taq™ II commercial kit (Takara, Dalian, China). The U6 gene was used as an internal reference. The relative expression of miR-211-5p was calculated by the 2^−ΔΔCt^ method.

### Follow-up plan and contents

According to the definition of cardiovascular endpoints issued by the American College of Cardiology [13] and the American Heart Association [10] in conjunction with the US Food and Drug Administration (FDA) and the Cardiovascular Trial Standard Data Collection Program (SCTI), death and hospitalization caused by events such as myocardial infarction, transient ischemic attack, stroke, acute heart failure, unstable angina, coronary interventional therapy and peripheral vascular intervention were defined as cardiovascular endpoint events. In this study, subjects were followed up for a fixed period of five years and the occurrence of cardiovascular endpoint events was recorded.

### Statistical analysis

The comparison among groups was analyzed by Student’s *t* test and one-way ANOVA. The diagnostic value of miR-211-5p for AS was evaluated by a receiver operating characteristic (ROC) curve. The correlations of continuous variables were analyzed by Spearman correlation coefficient. Kaplan–Meier curve and log rank method were used to evaluate the predictive ability of miR-211-5p on the occurrence of cardiovascular endpoint events in AS patients, and multivariate COX regression analysis was used to estimate the prognostic value of miR-211-5p in AS. The normality of experimental data was analyzed by Kolmogorov–Smirnov (K-S) normality test. The statistics and analysis of all data in this study were performed using SPSS 21.0 software (SPSS Inc., Chicago, IL) and GraphPad Prism 7 software (GraphPad Software, Inc., USA), and *P* < 0.05 was considered statistically significant.

## Results

### Clinical characteristics of subjects

The demographic characteristics and clinical data of all subjects in this study were shown in Table 1. The results showed that the level of diastolic blood pressure (DBP), C-reactive protein (CRP) and CIMT in AS patients were significantly higher than that in healthy controls (*P* < 0.05). However, there were no statistically significant differences between AS patients with healthy controls (*P* > 0.05) in age, gender, BMI, fasting blood glucose (FBG), total cholesterol (TC), low-density lipoprotein cholesterol (LDL), triglyceride (TG), systolic blood pressure (SBP), and high-density lipoprotein cholesterol (HDL).

**Table 1:** Clinical data of the study population

Paraments	Controls group (n = 85)	AS group (n = 90)	*P* value
N (Male and female)	(43/42)	(41/49)	0.505
Age (years)	63.71 ± 7.15	64.46 ± 7.30	0.494
BMI (kg/m^2^)	23.17 ± 2.96	22.89 ± 2.91	0.541
FBG (mg/dL)	88.56 ± 14.97	93.03 ± 17.47	0.071
TC (mg/dL)	192.47 ± 5.09	193.14 ± 3.77	0.321
TG (mg/dL)	122.62 ± 13.63	124.90 ± 13.86	0.275
HDL (mg/dL)	48.66 ± 4.52	47.75 ± 3.47	0.138
LDL (mg/dL)	114.73 ± 8.57	116.29 ± 6.95	0.188
SBP (mm Hg)	125.62 ± 7.61	127.67 ± 9.75	0.125
DBP (mm Hg)	73.99 ± 5.74	76.27 ± 5.66	0.009
CRP (mg/L)	3.35 ± 0.95	9.86 ± 2.16	< 0.001
CIMT (mm)	0.53 ± 0.17	1.02 ± 0.11	< 0.001

*BMI* body mass index; *TC* total cholesterol; *TG* triglycerides; *HDL* high-density lipoprotein; *LDL* low density lipoprotein; *SBP* systolic blood pressure; *DBP* diastolic blood pressure; *CRP* C-reactive protein; *CIMT* carotid intima-media thickness; *FBG* fasting blood glucose. Data are expressed as n or mean ± S.D

### Serum miR-211-5p expression levels in patients with AS

**Fig. 1:**
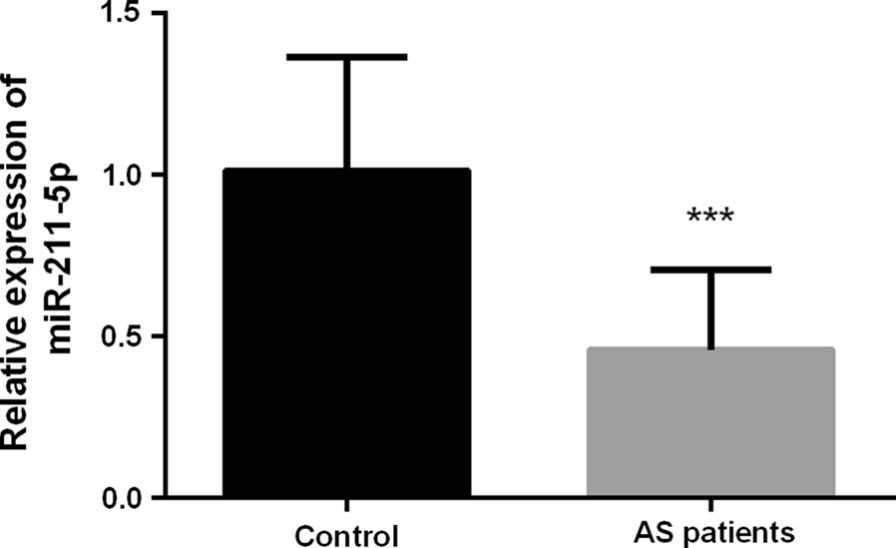
Serum miR-211-5p expression level in the study subjects. The expression level of miR-211-5p in AS patients was significantly decreased in comparison with the healthy controls. ***P < 0.001

The expression levels of miR-211-5p in serum of all subjects were measured by qRT-PCR technology. The result showed that the serum miR-211-5p expression level in asymptomatic AS patients was significantly decreased compared with healthy control groups (Fig. 1, *P* < 0.001).

### Correlation of miR-211-5p expression with CIMT and CRP in AS patients

**Fig. 2:**
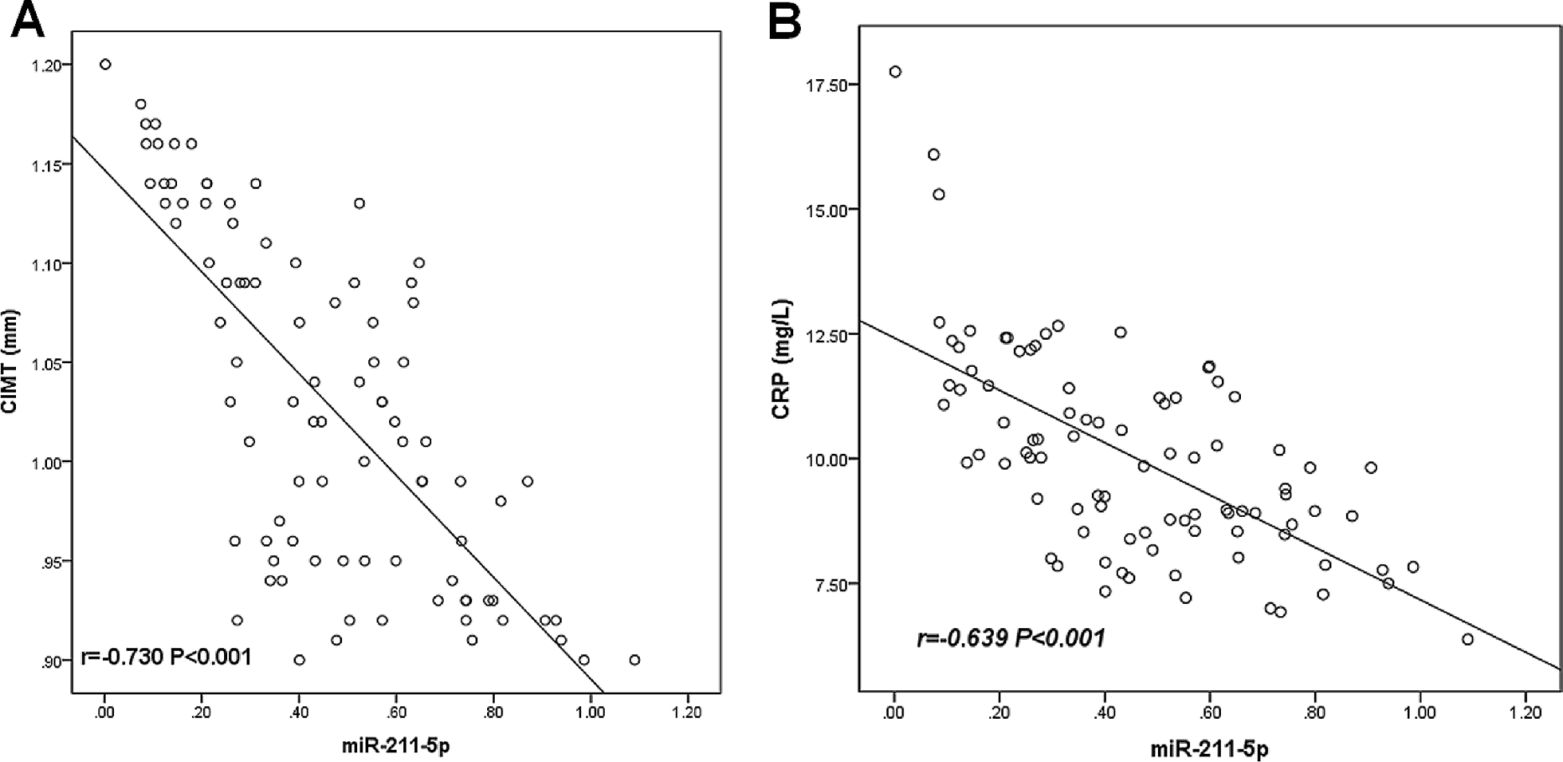
The correlation of serum miR-211-5p level with CIMT (**A**) and CRP (**B**) in AS patients. Serum miR-211-5p levels were negatively correlated with CIMT (r =  − 0.730, *P* < 0.001) and CRP (r =  − 0.639, *P* < 0.001) in AS patients

The earliest sign of AS is an increase in CIMT value and CRP level. In this study, Spearman correlation coefficient analysis was used to detect the correlation of miR-211-5p with CRP and CIMT. As shown in Fig. 2, miR-211-5p was highly expressed in patients with low CRP level and CIMT value, and miR-211-5p expression level was negatively correlated with CIMT (r =  − 0.730, *P* < 0.001) and CRP (r =  − 0.639, *P* < 0.001), respectively.

### Specificity and sensitivity of miR-211-5p as a diagnostic biomarker

**Fig. 3:**
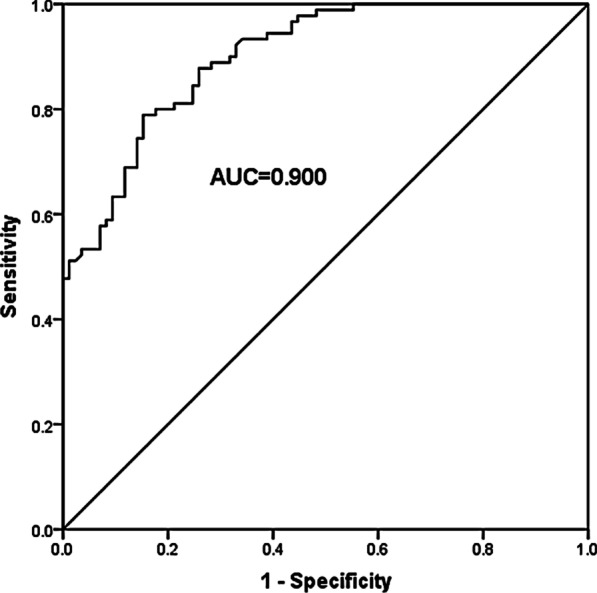
A ROC curve was established to evaluate the discriminability of serum miR-211-5p in diagnosing AS in the control groups. A high diagnostic power of miR-211-5p as a biomarker for AS was detected with an AUC value of 0.900

An ROC curve was established to evaluate whether miR-211-5p had the ability to distinguish AS patients from healthy control groups. We could see that the value of area under the curve (AUC) was 0.900 in Fig. 3, and the curve showed a sensitivity of 78.9% and a specificity of 84.7% at the cutoff value of 0.655, which suggested that miR-211-5p had a higher diagnostic value for AS.

### Kaplan–Meier survival curve analysis

**Fig. 4:**
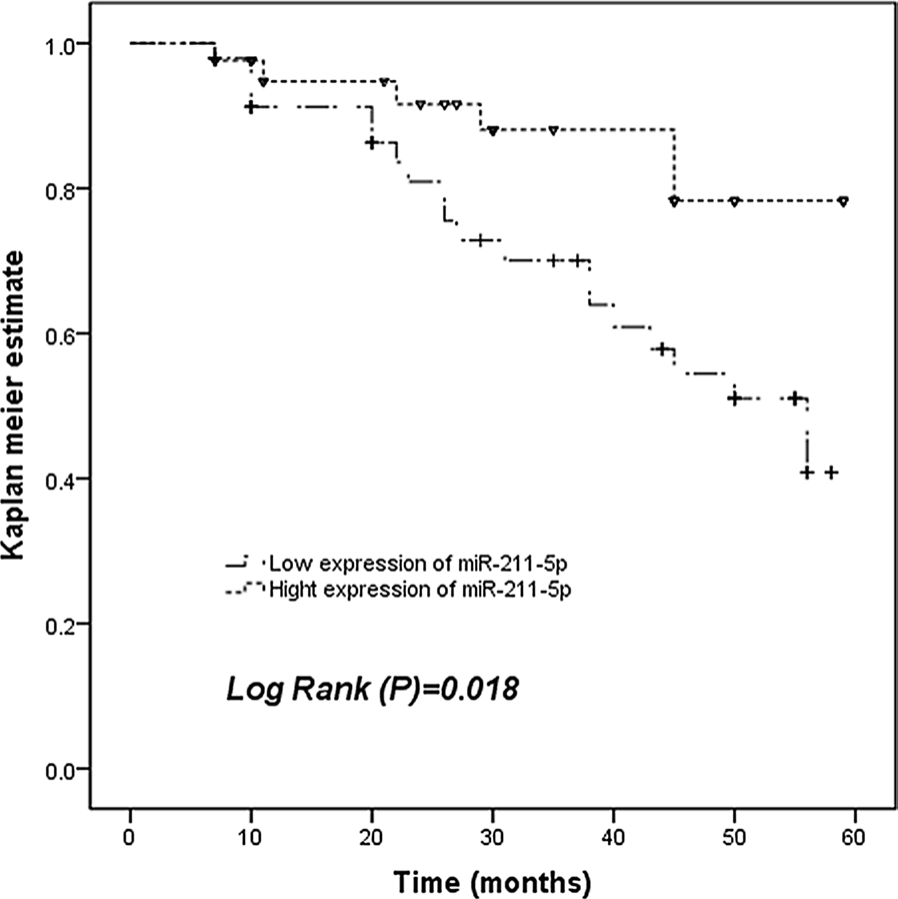
Kaplan–Meier curves of survival probability of patients with AS stratified by the serum miR-211-5p expression levels. Patients with low serum miR-211-5p levels had shorter event free survival probability than that with high serum miR-211-5p levels (Log Rank *P* = 0.018)

No subjects dropped out during the 5-year follow-up. According to the average expression level of miR-211-5p in the serum of AS patients, AS patients were divided into two groups: high expression group of miR-211-5p and low expression group of miR-211-5p. A total of 25 asymptomatic AS patients had cardiovascular endpoints, including 8 strokes, 3 myocardial infarction, and 14 transient ischemic attack, during the five-year period. Among them, 19 cases were from the group with low miR-211-5p expression, and the other 6 cases were from the group with high miR-211-5p expression. Then, the Kaplan–Meier survival curve was drawn using the data of the cardiovascular endpoint events. This curve demonstrated that the patients with low miR-211-5p expression level had shorter event-free survival probability than those who had higher miR-211-5p expression level (Fig. 4). Furthermore, in this study, we conducted further research and analysis on miR-211-5p with the clinical characteristics of patients through multivariate COX regression analysis. The results were shown in Table 2, miR-211-5p could be used as an independent prognostic factor for the occurrence of cardiovascular events in AS (HR = 0.324, 95% CI = 0.122–0.937, *P* = 0.038).

**Table 2:** Multivariate Cox regression analysis for the overall survival of AS patients

Paraments	Multivariate Cox regression analysis		
	95%CI	HR	*P* value
MiR-211-5p	0.122–0.937	0.324	0.038
Gender	0.534–3.820	1.428	0.478
Age	0.267–1.569	0.647	0.335
BMI (kg/m^2^)	0.466–2.742	1.130	0.786
FBG (mg/dL)	0.732–4.614	1.837	0.195
TC (mg/dL)	0.805–4.875	1.981	0.137
TG (mg/dL)	0.355–2.238	0.891	0.806
HDL (mg/dL)	0.245–1.727	0.651	0.388
LDL (mg/dL)	0.243–1.597	0.605	0.281
SBP (mm Hg)	0.552–3.240	1.337	0.520
DBP (mm Hg)	0.939–1.531	0.575	0.268
CRP (mg/L)	0.607–6.122	2.398	0.067
CIMT (mm)	1.009–6.551	2.571	0.048

*AS*:arteriosclerosis; *BMI* body mass index; *FBG* fasting blood glucose; *TC* total cholesterol; *TG* triglycerides; *HDL* high-density lipoprotein; *LDL* low density lipoprotein; *SBP* systolic blood pressure; *DBP* diastolic blood pressure; *CRP* C-reactive protein; *CIMT* carotid intima-media thickness

## Discussion

AS and its accompanying clinical complications, such as myocardial infarction, stroke, and peripheral artery disease, are still the main causes of morbidity and mortality in nowadays society. In the past few decades, miRNAs have emerged as evolutionary conserved non-coding small RNAs, and more and more studies have shown that miRNAs are very important in regulating key signal transduction and lipid homeostasis pathways [14, 15]. In the present study, it was found that the expression level of miR-211-5p in the serum of patients with AS was significantly lower than that of the healthy control groups, suggesting that the occurrence and development of AS may be related to the disorder of miR-211-5p.

Studies have found that a variety of miRNAs such as miR-34a, miR-217 and miR-146a can regulate the proliferation and differentiation of vascular endothelial cells, and may stimulate cell senescence, thereby triggering endothelial dysfunction [16, 17]. In the past ten years, the role of CRP in atherosclerotic cardiovascular disease has received increasing attention. Among the currently reported biomarkers, CRP may be the most promising indicator of vascular inflammation. CRP is an acute-phase protein, which is mainly produced when acute inflammation or infection occurs. Similarly, CRP can also be detected at the site of inflammation or injury [18, 19]. CIMT is an alternative indicator of the presence and progression of AS, and is used worldwide because of its simplicity, repeatability, and non-invasive [20]. The study of Bots et al. showed that the CIMT value of patients with aortic plaque was 0.12 mm, which was much higher than that of patients without plaque [21]. In this study, through the detection of the expression level of miR-211-5p and the measurement of serum CRP and CIMT, we found that the expression level of miR-211-5p in AS patients was significantly lower than that in healthy controls. And miR-211-5p showed negative correlation with CRP and CIMT. This result preliminarily indicated that miR-211-5p might be associated with the occurrence and development of AS.

More and more studies emphasized the importance of miRNAs in the development and progression of AS. In recent years, studies have found that miRNAs play a crucial role in the pathophysiology of AS by regulating arteriosclerotic genes and regulating post-transcriptional gene expression. Therefore, by influencing the levels of synthetic proteins in cells, they may play a role in driving disorders that affect endothelial cells, smooth muscle cells, and white blood cells, thereby initiating and enhancing the growth of atherosclerotic plaques. There is increasing evidence that the impact of genes on AS allows us to use miRNAs as new treatments or clinical biomarkers to better manage cardiovascular disease [22–24]. Mohammad et al. confirmed that in the peripheral blood of patients with multiple sclerosis (MS), the expression levels of miR-211-5p and miR-34a-5p were significantly down-regulated compared with the healthy controls [25], suggesting that MS may be caused by these genes regulatory abnormalities. This evidence supported our research. In our study, a ROC curve was used to assess the diagnostic significance of miR-211-5p for AS, and results showed that miR-211-5p had high sensitivity and specificity and low expression of miR-211-5p could be used as a biomarker for diagnosis of AS. Furthermore, data from a five-year follow-up of AS patients showed that the prognosis of patients with low miR-211-5p expression was poor, and data from multivariate COX regression analysis also proved that miR-211-5p was an independent prognostic factor for the occurrence of cardiovascular events in AS. Together, these data confirmed that patients with low miR-211-5p expression have a higher risk of cardiovascular endpoint events.

In current times, miR-211-5p, 22 nucleotides in length, are located on human chromosome 15 [26]. It was found that miR-211-5p inhibited the proliferation, invasion, migration, and metastasis of triple negative breast cancer tumor cells by directly targeting SET Binding Protein 1 (SETBP1) [27]. In another study, miR-211-5p played an important role in Alzheimer's disease (AD) through regulating neuronal differentiation and viability [28]. As Sara et al. reported, miR-211-5p is involved in the regulation of vascular smooth muscle cell calcification [11]. According to the above analysis, this study proved that miR-211-5p is low expressed in the serum of AS patients, and low expression of miR-211-5p was related to poor prognosis of AS. Combined with the previous research and the present results, we speculated that miR-211-5p might be involved in the development of AS via regulating vascular smooth muscle cell behaviors. But further studies are needed to verify the hypothesis.

Nevertheless, some limitations are present in this study. On the one hand, the sample size of this study was small. TC, LDL, and other important indicators of AS showed no significant difference between the two groups of study population. Meanwhile, the effect of miR-211-5p on blood lipid indexes could not be determined. Furthermore, CRP also played a crucial role in AS. Based on the current results, we cannot determine whether inflammation is a cause or a result of AS. Therefore, this still requires us to expand the sample size for further verification. On the other hand, the regulation of miRNA is mainly accomplished by binding to downstream mRNA, thereby inhibiting mRNA translation or promoting mRNA degradation. However, this study only evaluated the diagnostic and prognostic value of miR-211-5p in AS and did not investigate the relevant mechanism. In conclusion, we preliminarily determined that miR-211-5p can be used as a new biomarker for the diagnosis of AS, and the low expression of miR-211-5p is associated with the poor prognosis of AS. Although we have confirmed through the above experimental data that there is indeed an abnormal regulation of miR-211-5p in patients with AS, the molecular mechanism of the phenomenon still needs to be further studied.

## Data Availability

The datasets used and/or analysed during the current study are available from the corresponding author on reasonable request.

## Abbreviations

ACC: American College of Cardiology
AHA: American Heart Association
AS: Atherosclerosis
AUC: Area under the curve
BMI: Body mass index
CIMT: Carotid intima-media thickness
CRP: C-reactive protein
DBP: Diastolic blood pressure
FBG: Fasting blood glucose
FDA: Food and Drug Administration
HDL: High-density lipoprotein cholesterol
LDL: Low-density lipoprotein cholesterol
miRNAs: MicroRNAs
ROC: Receiver operating characteristic
SBP: Systolic blood pressure
SCTI: Standard Data Collection Program
SETBP1: SET Binding Protein 1
TC: Total cholesterol
TG: Triglyceride
